# Flexoelectricity, Triboelectricity, and Free Interfacial Charges

**DOI:** 10.1002/smll.202310546

**Published:** 2024-08-25

**Authors:** L. D. Marks, K. P. Olson

**Affiliations:** ^1^ Department of Materials Science and Engineering Northwestern University Evanston IL 60208 USA

**Keywords:** contact electrification, displacement field, flexoelectricity, triboelectricity

## Abstract

Triboelectricity has been a topic of some confusion for many years, probably because it is very diverse and some of the fundamental science has not been clear. This is now starting to change. A few years ago, the importance of flexoelectricity at asperities is pointed out. That paper exploited the established physics of compensation of bound surface or interfacial charges without going into detail. The purpose of this paper is to expand further on this, mapping from the established physics of electrostatics with contact potentials and Maxwell's displacement field to the underlying fundamentals of charge transfer in triboelectricity. Examples from the published literature are used to illustrate this. In the discussion, some of the open questions and challenges to the community are mentioned.

## Graphical abstract

What is observed macroscopically for triboelectric charging as shown on the left involves polarization, bound surface charges, and free charges as shown on the right.

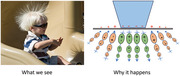

## 1 Introduction

Whenever two insulators (and sometimes metals) rub together, or particles in powders collide, static electricity is generated, a phenomenon called either contact electrification, if there is no sliding, or triboelectricity in the more general case. It was recorded by Thales of Miletus around 585 BC^[^ ^1^ ^]^ after rubbing amber with fur, although it may have been known earlier.^[^ ^2^ ^]^ The word has roots in the Greek words *tribo*, to rub, and *ēlektron* for amber.

Static electricity affects our daily lives and also impacts many technologies, from stroking a cat and combing human hair,^[^ ^3^, ^4^ ^]^ to problems during the processing of pharmaceutical powders,^[^ ^5^ ^]^ refinement of wheat bran,^[^ ^6^ ^]^ as a method for nanoscale energy harvesting,^[^ ^7^ ^]^ how electric charges develop in blowing sand, snow or volcanic plumes,^[^ ^8^, ^9^ ^]^ as a key element in how millimeter size grains aggregate during planetary formation,^[^ ^10^ ^]^ and changes in plant physiology such as plant sap flow due to the electric charges on sand during desertification^[^ ^11^ ^]^ – an incomplete list. While most commonly found for solid‐solid contacts, triboelectric charging can occur in other cases, for instance the early work of Faraday in 1843 for water droplets sliding on materials,^[^ ^12^ ^]^ the classic waterfall effect analyzed by Lenard in 1892,^[^ ^13^ ^]^ and current safety issues with pneumatic transport systems.^[^ ^14^ ^]^ Static electricity is to blame for at least two serious fires or explosions *each day* in manufacturing plants worldwide.^[^ ^15^ ^]^

There is a large amount of good science in earlier works that has relevance to aspects of this paper, so a brief review to highlight some of this is appropriate; much of the early work has been reviewed by Roller and Roller.^[^ ^2^ ^]^ The first major scientific analysis was by Gilbert in 1600.^[^ ^16^ ^]^ He discovered that rubbing more materials than just amber such as sulphur, wax, and glass could produce static electricity. Others such as Browne in 1672^[^ ^17^ ^]^ observed that metals did not show triboelectric charging, presumably because they conducted charge to ground. One of the more significant early papers was by Wilcke in 1757 who proposed a triboelectric series^[^ ^18^ ^]^ where materials towards the bottom of the series would charge negatively compared to ones higher up. While this approach has some justification in terms of contact potentials (work function differences), it fails in many cases, for instance with identical materials; however, it remains a common approach in the literature.

The first systematic analysis of triboelectricity was by Péclet in 1834,^[^ ^19^ ^]^ a more scientific analysis. Important herein is the work of Harris in 1867,^[^ ^20^ ^]^ who mentions in Chapter 1 that the sign of charging can depend upon how much pressure is used, a result that has only very recently been explained as we will see later. The most extensive early set of experimental analyses was from 1914–1930 by the group of Shaw, who laid much of the foundation of experimental knowledge including the failing of the triboelectric series and the role of heat;^[^ ^21^ ^]^ described many cases for charging between different materials;^[^ ^22^, ^23^, ^24^, ^25^ ^]^ and demonstrated that surface strain mattered.^[^ ^26^, ^27^ ^]^ Many of his results have only recently started to be fully understood, and are relevant herein.

Most of the early work predates the modern description of bulk and surface band structure and concepts such as band‐bending and Fermi level pinning at surfaces and interfaces. It was in the early 1950s, in the work of authors such as Vick,^[^ ^28^ ^]^ Montgomery,^[^ ^29^ ^]^ and Harper^[^ ^30^ ^]^ that these were first taken into account, along with processes such as quantum tunneling and Schottky barrier effects, and also modern approaches to asperity contacts based upon the work of Bowden and Tabor.^[^ ^31^ ^]^ For instance, as early as 1958 Levy, Wakelin, Kauzmann and Dillon^[^ ^32^ ^]^ qualitatively connected asperity‐asperity contacts to triboelectric charging.

That elastic deformation can change charge transfer has been known since the early 20^th^ century. Definitive evidence for the importance of flexoelectricity in triboelectricity can be found in the work of Jamieson in 1910,^[^ ^33^ ^]^ who showed that changing the bending of a piece of cellulose changed the sign of the tribocharge. This was extended by Shaw in 1917^[^ ^22^ ^]^ who explored more materials, demonstrating that some charged more positively with positive curvature, while others charged more negatively. There is also other work demonstrating that strain can matter, from the early work of Shaw and Hanstock^[^ ^26^ ^]^ to more recent from the group of Lacks.^[^ ^34^ ^]^ Other related work shows that surface finish matters, ranging from the 1867 work of Harris^[^ ^20^ ^]^ who notes differences between polished and rough glass, to more recent measurements of polarity changes in the contact electrification of polyethylene by Ge et al^[^ ^35^ ^]^ and charge transfer of statistical surfaces as modeled by Persson^[^ ^36^ ^]^ and measured by Zhai et al.^[^ ^37^ ^]^

The early (and more recent) work indicates that there are several key terms that need to be considered:

1. Work function differences between the two materials, also called the contact or Volta potential.
2. Local curvature, strain, and roughness.
3. The electronic structure of the materials, and the crystallography of the contacting facets.
4. The forces during contact/sliding, and the velocities when particles collide.
5. Surface and interface states, including traps.
6. Environmental factors, such as humidity and temperature.

**Figure 1:**
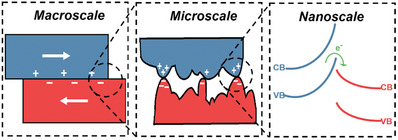
From the left to the right, with two contacting or sliding surfaces at the macroscale there are asperities of different radii and crystallographic contacts at their tips at the micro scale which, in turn, connects to different contact potentials and band bending.

How do we combine all of these? We recently introduced the idea that the flexoelectric polarization due to asperity contacts could play an important role.^[^ ^38^, ^39^ ^]^ The flexoelectric effect, the polarization caused by strain gradients, is large at the nanoscale because compared to other terms such as piezoelectricity, the gradient introduces a scaling that is inverse with feature size. Formal flexoelectric theory dates back to the 1960s,^[^ ^40^, ^41^ ^]^ and a number of reviews discuss details further.^[^ ^42^, ^43^, ^44^, ^45^ ^]^ As illustrated in **Figure** **1**, with two macroscopic contacting surfaces, the contacts are microscopic to nanoscale asperities within which there is local band bending.

In the original paper^[^ ^39^ ^]^ we argued that, to quote:

“the triboelectric surface charge density is set by the flexoelectric polarization, i.e., charge will transfer until the flexoelectric polarization is screened”.

This was used in one comparison with experimental results, but not expanded upon. The intention of this note is to expand on this statement using standard electrostatics where the polarization is reduced to a bound charge density and external electric fields such as the contact potential between dissimilar materials. We show that handled this way, the first four of the key terms listed above fit into an approach which is consistent with standard interface physics, without any need to introduce ad‐hoc or unverifiable additional terms. The boundary condition on the displacement field density at the interfaces correlates with the interfacial free charge which constitutes the maximum possible triboelectric charge transfer. At the same time, we will describe some of the experimental evidence by others that also point to the importance of the flexoelectric effect in triboelectricity. We will return in the discussion to some of the open questions and propose challenges for the community.

## 2 Capacitor‐Like Compensation and Surface Bound Charge

**Figure 2:**
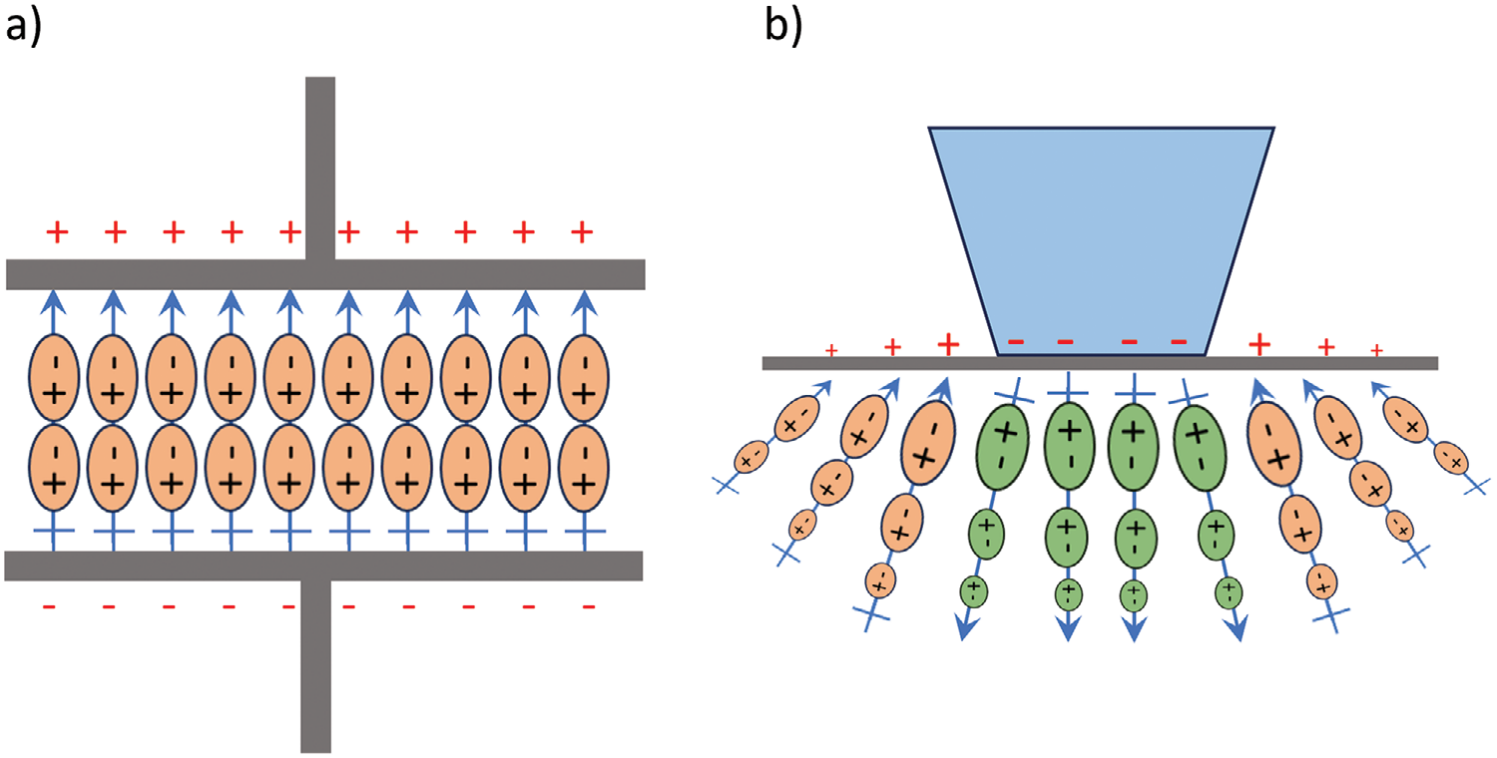
In a) representation of a capacitor with dipoles (brown ovals) for the polarization in the dielectric. To balance the bound charges at the top and bottom there will be additional charges on the top and bottom plates. The total charge will be the sum of the charges that balance the applied potential between the plates and the surface‐bound charge. In b) representation of the polarization due to an asperity contacting a surface, with the flexoelectric polarization decaying with distance and surface charges to compensate the bound surface charges due to the polarization.

A useful way to look at the fundamentals of charge transfer is analogous to how a capacitor works. The underlying physics is well established but has often been overlooked or misunderstood. Consider a simple parallel plate capacitor with a dielectric between the two plates as sketched in **Figure** **2**a. The polarization within the dielectric is represented by the small orange dipoles, which for the case drawn, are net negative at the top surface and net positive at the bottom, see below for more rigor. To balance the polarization there will be positive and negative charges on the grey metal plates. The total charge on the plates will be the sum of the contributions due to the applied potential across the capacitor plates and that in the dielectric.

This can be compared to the polarization due to elastic deformations around asperity contacts,^[^ ^39^, ^46^ ^]^ as schematically represented in Figure 2b. There will again be charges at the top surface to balance the net polarization; for a thick enough substrate, the polarization will decay to zero inside the dielectric far from the outer surface, about ten times the contact radius of an asperity or a few times the effective radius of an asperity.^[^ ^46^ ^]^

Being more rigorous, there is a standard decomposition of the polarization density **P**(**r**) as a function of position **r** into what are called “bound charges”.^[^ ^47^ ^]^ For a given polarization, the potential *V*(**r**) is given by the integral over all space:

(1) $$V\;\left(r\right)=\frac{1}{4\pi \varepsilon }\;\int_{\Omega }P\left(r^{\prime }\right)\cdot \frac{r-r^{\prime }}{{\left|r-r^{\prime }\right|}^{3}}dr^{\prime }$$

where ε is the permittivity. Combining this with the divergence theorem and integration by parts, this can be transformed to

(2) $$V\;\left(r\right)=\frac{1}{4\pi \varepsilon }\;\int_{\Omega }-\frac{\nabla^{\prime }\cdot P\left(r^{\prime }\right)}{\left|r-r^{\prime }\right|}dr^{\prime }+\frac{1}{4\pi \varepsilon }\;\oint_{S}\frac{P\left(r^{\prime }\right)\cdot n\left(S^{\prime }\right)}{\left|r-r^{\prime }\right|}dS^{\prime }$$

with **n**(**S**′) the surface normal, the integral *dS*′ being over the surface. This can be written more simply as the sum of a bound bulk charge density **ρ**_**b**_(**r**) and bound surface charge density **ρ**_**s**_(**r**)

(3) $$V\;\left(r\right)=\frac{1}{4\pi \varepsilon }\;\int_{\Omega }\frac{\rho_{b}\left(r^{\prime }\right)}{\left|r-r^{\prime }\right|}dr^{\prime }+\frac{1}{4\pi \varepsilon }\;\oint_{S}\frac{\rho_{s}\left(r^{\prime }\right)}{\left|r-r^{\prime }\right|}dS^{\prime }$$

The bound charges cannot move around; they are fixed by the polarization. However, the surface polarization cannot be “naked”, it must be compensated. For instance, using the standard analysis given by Tasker^[^ ^48^ ^]^ the potential energy is nominally singular if the polarization is uncompensated. (There are some issues related to surface polarization which, for oxide surfaces, are still not fully settled in the literature, see;^[^ ^49^, ^50^, ^51^ ^]^ we will return to some of these in the discussion). If we assume that atomic positions do not significantly change during the elastic deformation, then some mobile charges must come from elsewhere to avoid singular surface energies, these mobile charges are commonly called free charges. For completeness, these could also be ions.

Note that this form also arises with the displacement field in Maxwell's equations in matter, also sometimes called Maxwell's macroscopic equations. In a Maxwellian approach, see,^[^ ^47^, ^52^, ^53^ ^]^ the electric displacement field **D** is introduced via

(4) $$D\;=\varepsilon_{0}\;E+P$$

where ɛ_0_ is the vacuum permittivity and **E** the electric field due to both external potentials and any free charges. The general form of Ampère's law is then

(5) $$\nabla \cdot D\;=\varepsilon_{0}\nabla \cdot E+\nabla \cdot P=\rho_{f}\;$$

where ρ_*f*_ is the free charge density, for instance electrons or ions. Applying the condition that the displacement field is zero outside a solid would then give a boundary condition:

(6) $$D\cdot n\;=\varepsilon_{0}\;E\cdot n+P\cdot n\;=\rho_{f}^{S}\;$$

with $\rho_{f}^{S}$ the surface free charge density and, as before, **n** is the surface normal. This equation is evaluated at the surface. Note that just as **P** · **n** can be thought of as a bound surface charge from the polarization, there is also an effective bound surface charge ɛ_0_ **E** · **n** from the electric field. Also note that the surface here may be the interface between two materials. If there is no free surface charge then the boundary condition is:

(7) D·n=0

which would be the relevant boundary condition for a free surface in isolation. In the absence of any elastic deformation, the contact potential and associated band bending as well as any surface dipoles are the only terms that matter; when there is elastic deformation the electromechanical terms such as piezoelectricity and flexoelectricity also contribute. Since within a metal in equilibrium there is no electric field or polarization, for a metal‐insulator or metal‐metal contact we would use the boundary condition (6) with $\rho_{f}^{S}$ the interfacial charge density.

The above descriptions are, more formally, what was mentioned in our earlier work.^[^ ^39^ ^]^ For completeness, in a triboelectric nanogenerator, with 100% efficiency, the current would be the change in the free carrier density at the interface with time. This is an upper limit, in practice, there will be loss mechanisms where not all the current is collected.

The sections above are standard textbook material, and the interpretation of polarization as equivalent to a bound surface charge can perhaps be traced back to the original work of Maxwell,^[^ ^54^ ^]^ see the 1951 analysis of Fisher^[^ ^53^ ^]^ for more details. When discussing tribocharge sometimes the textbook derivations have been rederived.^[^ ^55^, ^56^, ^57^ ^]^ Unfortunately they have also been misinterpreted. For instance, it has been suggested that an additional surface polarization term needs to be added,^[^ ^58^ ^]^ which is incorrect as it is already present in the boundary conditions. There are also other approaches^[^ ^57^, ^59^ ^]^ where the displacement current density, the time derivative of the displacement field density, has been equated to the free‐carrier current without fully including the boundary conditions. While there are similarities, as Maxwell himself pointed out, they are not the same. Whether the charge compensating boundary condition of Equation (6) matters or the zero free surface charge of Equation (7) should be used or a combination of the two is a subtle issue that we will return to in the discussion.

## 3 Experimental Evidence

We will now review a range of different results which cleanly establish the relationship between the compensation mechanism we originally described and details in the previous section. Several of these also involve small‐scale contacts where flexoelectric contributions become important, particularly for nanoscale contacts.

## 4 Classic Experiments

**Figure 3:**
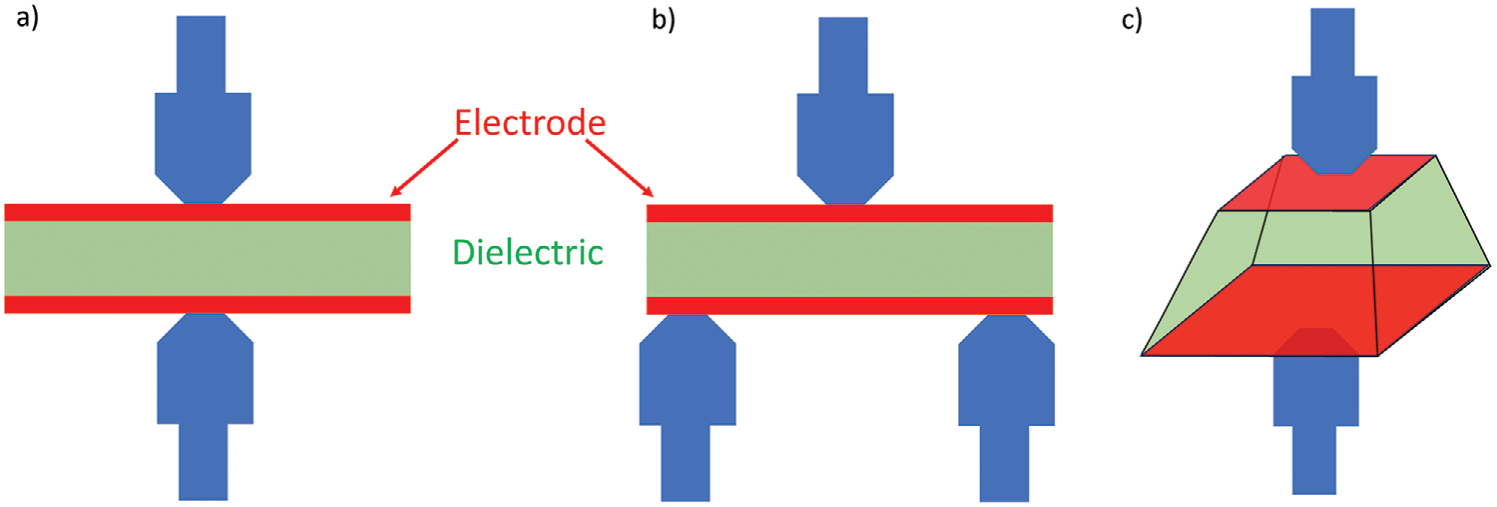
In a) the Belincourt method for a piezoelectric where an oscillating force is applied on the two blue clamps, and the charge is measured on the red electrodes. In b) the standard method to measure flexoelectric coefficients using a three‐point bending; everything else is essentially the same as in the Belincourt method. In c) the pyramid compression method used to measure flexoelectric coefficients, which is comparable to an asperity under normal load.

The simplest experiments to validate the surface bound charge compensation approach are those where the bulk piezoelectric coefficient is measured using what is typically called the Berlincourt method^[^ ^60^ ^]^ as illustrated in **Figure** **3**a; see^[^ ^61^, ^62^, ^63^, ^64^ ^]^ for some examples of experimental measurements. An oscillatory force is applied to a piezoelectric material that leads to elastic deformations that are as linear as possible, then the charge on the plates is collected. Related to this is the classic approach to measure flexoelectric coefficients, where one of the most common methods uses three‐point bending instead of a linear strain as illustrated in Figure 3b, see refs.[43, 65, 66, 67] for examples of this approach. Note that while both of these are bulk experiments, the extension to a nanoscale experiment where the probe is an asperity is somewhat direct, with the bottom electrode being replaced by a semi‐infinite bulk.

Of major relevance for later, an alternative way to measure flexoelectric coefficients is by using the compensating charge on the top and bottom plates of a deformed pyramid, a standard approach^[^ ^43^, ^68^, ^69^, ^70^ ^]^ as illustrated in Figure 3c. Note that this is a macroscopic version of a single asperity contact under normal load.

There are also inverse cases, where an applied voltage or charge is used to induce linear strain for electrets or bending due to the flexoelectric effect. For bending, this is how the first flexoelectric experiments were performed by Bursian and Zaikovskii.^[^ ^41^ ^]^ The converse effect where asymmetric bending during charging inside a transmission electron microscope has also been directly observed.^[^ ^71^ ^]^

## 5 Single Asperity Experiments

**Figure 4:**
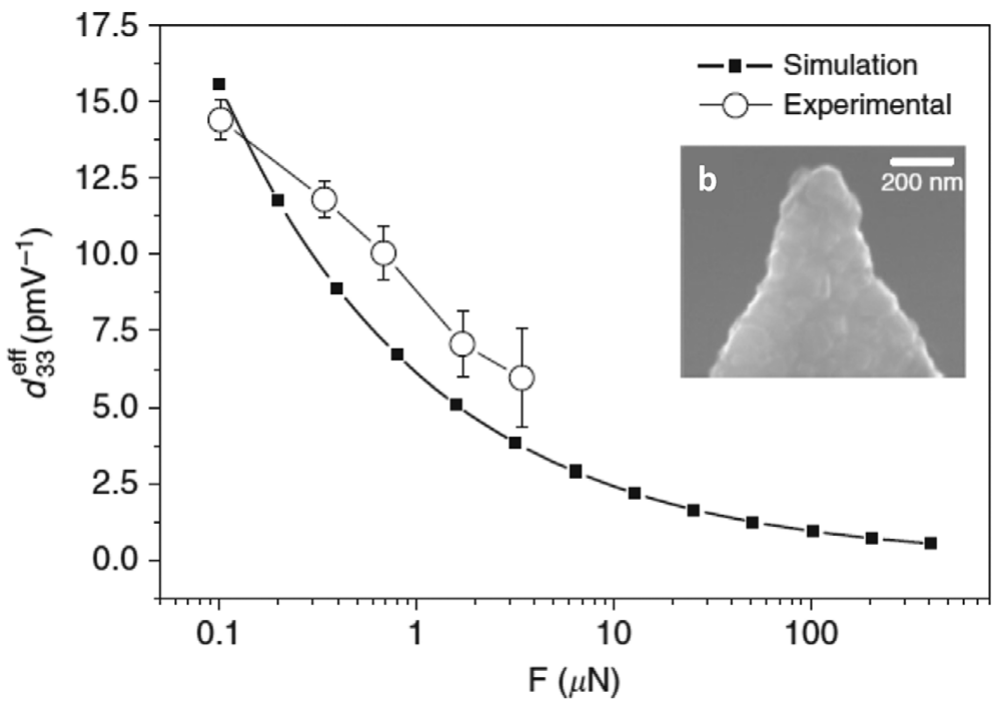
Converse flexoelectricity induced at the tip apex of an atomic force microscope cantilever as a function of the applied force. In a) the effective piezoelectric coefficient as a function of applied force for a SrTiO_3_ crystalline substrate. Filled squares correspond to the values obtained by a simulation. Empty circles correspond to the experimental values with a Nanosensors CDT FM tip and a cantilever of medium stiffness (k ≈ 2.8Nm^−1^) coated with doped diamond. The error bars correspond to the error of the linear fitting of the experimental data, which compares the measured electromechanical amplitude of oscillation Δh with the Vac applied voltage. In b) the effective contact radius “a” scales with the force and is determined by the tip radius. The experimental tip radius was measured from scanning electron microscopy images of the used tip. The tip radius of the diamond‐coated tip was 105 nm and remained spherical shape after the measurements. Reproduced from the Open Access article by Abdollahi et al,^[^ ^75^ ^]^ Creative Commons Attribution 4.0 International License.

The cleanest connection between the classic experiments detailed above and ones involving more general systems is single asperity experiments; as will be discussed later, the case with multiple asperities is less well‐defined. One set of these is what could be called “inverse triboelectricity”, displacements due to applied voltages. There is extensive literature on these with piezoelectric materials, for instance, references.^[^ ^72^, ^73^, ^74^ ^]^ More recently the deformation associated with the converse flexoelectric effect where a sample strains due to the application of an applied voltage has been measured and modeled by Abdollahi et al, see **Figure** **4**.^[^ ^75^ ^]^ These authors converted the surface displacement into an effective piezoelectric component as a function of the applied force, using as an electric boundary condition in their simulations that the normal component of the displacement field is zero, see Equation 7. The agreement of the calculations and experiment is quite good. The opposite of this, changes in potential with applied strain which is closer to triboelectricity, has been measured quantitatively by us,^[^ ^76^ ^]^ with a more detailed analysis of the effect of asperity shape published in early 2024.^[^ ^46^ ^]^ For completeness, there has also been recent work that has also looked at band‐bending due to flexoelectricity at the nanoscale,^[^ ^44^, ^77^, ^78^, ^79^ ^]^ although they have not always made the next step to consider the displacement field boundary conditions and charge transfer.

## 6 Macroscopic Experiments

Some care is needed to translate from single asperity contacts to multiple contacts. It is established that when two general bodies contact it is via a number of asperities.^[^ ^31^ ^]^ The macroscopic laws of friction are a consequence of statistical changes in the number and area of contact, rather than being a fundamental property of a single contact. A common approach (e.g.,)^[^ ^80^ ^]^ is to consider that the load is carried by asperities which are just at the plastic yield strength – and with a higher stress any given asperity would deform. The total contact area will then be the applied load divided by the yield strength, which is often a small fraction of the apparent macroscopic contact area. This means that the conversion from single asperity to multiple contacts is complex.

**Figure 5:**
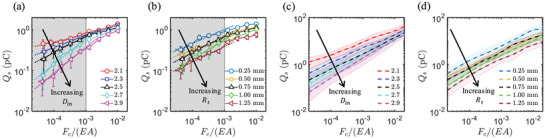
In a) and b) experimental results, and c) and d) modeling for charge transfer of fractal rough surfaces. In all cases, the x‐axis is the applied force F_c_ scaled by the Youngs modulus E and the apparent, i.e., macroscopic area A. The ratio F_c_/E is proportional to the true contact area. In all cases, the y‐axis is the charge transfer. In a) and c) the fractal dimension changes; small values mean less variation in the roughness. In b) and d) the roughness amplitude changes. Adapted from Zhai et al^[^ ^37^ ^]^ with permission from Elsevier.

The behavior for multiple flexoelectric contacts has recently been analyzed in detail by Zhai et al.^[^ ^37^ ^]^ These authors 3D printed rough surfaces with measured roughness distributions. Assuming an elastoplastic model for each asperity under normal loading, they then extracted the flexoelectric polarization as a sum over asperities of different radii and matched the charge on an effective micro contact area as that which will balance the polarization, similar to other work herein. Their results indicate the complexity of dealing with real surfaces rather than simplified flat surfaces or single contacts. Some of their results are shown in **Figure** **5**. They find:

1. The charge transfer scales with the nominal pressure, the force per macroscopic area, which is proportional to the true contact area.
2. The results depend upon both the fractal dimension, reduced charge for higher dimension, and decrease with increasing roughness.

They conclude that the charge transfer tends to be concentrated on the larger microcontacts, and scale with the true contact area. These results have implications for other macroscopic triboelectric tests and probably merit further investigation using charge harvesting devices.

**Figure 6:**
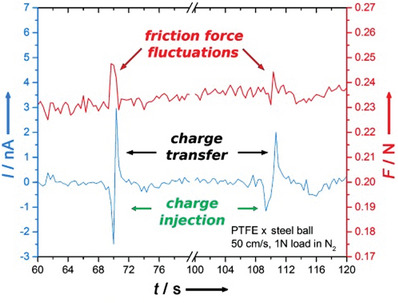
Friction force fluctuations and tribocurrent generation at the metal–PTFE interface, reproduced with permission from Bursan and Erdemir^[^ ^81^ ^]^ with permission from John Wiley and Sons.

A related experiment which also connects to flexoelectricity was performed by Burgo and Erdemir^[^ ^81^ ^]^ who investigated more macroscopic tribometry experiments coupled with charge measurements for a steel ball on PTFE. They observed that there were two processes taking place: charge transfer from the steel ball to the PTFE during compression followed by a backflow of charges from the PTFE to the metal surface during pull‐off, the two processes associated with a temporary friction force increase, see **Figure** **6**. While the contact potential will depend only weakly upon strain via the deformation potential, the main electromechanical terms whether piezoelectric or flexoelectric will change signs between compression (contact) and tension (pull‐off). This sign reversal is consistent with their observations.

## 7 Curvature Dependence

In many ways the most definitive proof of the role of curvature, that is flexoelectricity as an electromechanical contributor to the polarization and tribocharge, is the original work of Jamieson in 1910 when sliding two pieces of celluloid against each other, and in later experiments two pieces of paper, vulcanite, and shellac. He phrases it very well:

“Of the surfaces in contact, one is in compression, the other in tension; with celluloid, the compressed surface is always negatively, the stretched surface positively, electrified.”

**Figure 7:**
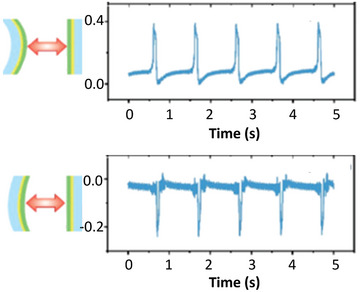
At the top, voltage using a triboelectric nanogenerator (TENG) when a convex piece is brought into contact with a flat piece of the same material, and bottom when a concave piece is. Adapted with permission from Figure 2e and 2i of Xu et al,^[^ ^82^ ^]^ copyright 2019 American Chemical Society.

These results were confirmed by Shaw in 1917.^[^ ^22^ ^]^ Effectively the same experiment has recently been performed by Xu et al,^[^ ^82^ ^]^ who it appears were not aware of the earlier work. These authors contacted two pieces of nominally the same material, one flat and the other curved either concave or convex. As expected, the sign of the charge transfer changed with curvature as shown in **Figure** **7**.

## 8 Sign Variations

It is worth quoting from the 1867 work of Harris:^[^ ^20^ ^]^

“If we strike a feather lightly against dry canvas, it becomes electrified negatively; if drawn with pressure between folds of the same canvas, it is electrified positively.”

This type of variation was also noted by Shaw,^[^ ^22^ ^]^ and has been modeled in some detail by Mizzi and Marks.^[^ ^38^ ^]^ When the forces are small the contact potential between two materials dominates; remember that the contact potential corresponds to the difference in the Fermi energy at the interface between the two materials if no charge transfer is allowed to occur. As the forces are increased there will be local band bending so the relative positions of the Fermi energies in the two materials can switch. In this case, there can be Zener tunneling^[^ ^83^ ^]^ and charge transfer in the opposite direction.

**Figure 8:**
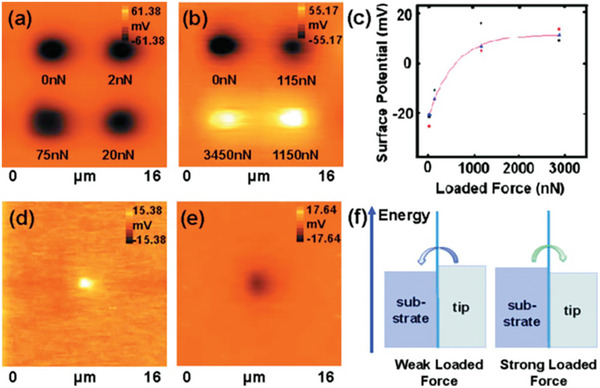
a) and b) Potential images obtained by using single crystal silicon tips on SiO_x_ substrates. a) CSG11 cantilever and b) NSG11 cantilever. The corresponding forces are shown under each charge pattern. c) Force dependence of surface potential when using NSG11 tips scanned over a SiO_x_ surface. The squares, the triangles, and the dots show three groups of parallel experimental data. d) and € Potential images of contact experiments obtained applying different forces on the NSG11 tip. d) 3450 nN, and e) 115 nN. f) Schematic of charge sign reversal model. The arrowhead shows the direction of the electron flow. Reprinted from Sun et al.^[^ ^84^ ^]^ with the permission of AIP Publishing.

An elegant demonstration of this is the work of Sun et al.^[^ ^84^ ^]^ These authors used single crystal silicon tips on SiO_x_ substrates with both a CSG11 cantilever and an NSG11 tip, with the results shown in **Figure** **8**. The spring constant of the NSG11 cantilever is about a hundred times larger, so much larger forces are being applied. With the CSG11 cantilever (low force) the authors observed increasing charge transfer with force; with the NSG11 (high force) they observed a reversal of the sign. The authors gave an explanation in terms of changes in the Fermi energies but did not connect this to electromechanical effects. In a later paper by some of the same authors using a conductive diamond tip on TiO_2_ the change in sign was attributed to flexoelectricity.^[^ ^85^ ^]^

## 9 Discussion

We presented some of the evidence that a conventional approach where the contact potential between two materials combined with additional polarization terms such as those from electromechanical effects helps explain contact and triboelectric charge transfer. The relevant boundary condition involves the normal component of the Maxwellian displacement field. We do not claim that we have included all the experimental and theoretical data, just a few key ones which we believe are definitive. We have also deliberately not dwelled on our own results,^[^ ^38^, ^39^, ^46^, ^65^, ^76^ ^]^ instead presenting mainly evidence from others.^[^ ^37^, ^44^, ^67^, ^68^, ^70^, ^75^, ^77^, ^78^, ^79^, ^81^, ^82^, ^84^, ^85^ ^]^ We have focused on experimental reports where the results are quite definitive, as against including ones which circumstantially support the role of displacement fields and charge compensation, of which there are many. While some of the results included are relatively recent, we have included some that date back to the early 20^th^ century or even earlier,^[^ ^12^, ^13^, ^16^, ^17^, ^18^, ^19^, ^20^, ^21^, ^22^, ^23^, ^24^, ^25^, ^26^, ^27^ ^]^ believing that it is important to appreciate that some of the evidence is not new. We suspect, albeit cannot prove that if a better understanding of electromechanical terms such as flexoelectricity existed in the early years of the 20^th^ century, much more would be settled about the fundamental science of contact and triboelectric charge transfer.

The classic Maxwellian approach with electromechanical terms and boundary conditions defines the compensating free carriers, but some care is needed, and it is not necessarily the same as the final state. For metal‐metal contacts, the equilibrium final state will be with surface charge (free carriers) balancing the contact potential. This connects with numerous experiments under relatively well‐controlled conditions and the importance of the contact potential which is also called the Volta potential; see for instance the discussion in his lecture by Helmholtz in 1881,^[^ ^86^ ^]^ early work by Harper in 1951^[^ ^87^ ^]^ and others more recently.^[^ ^88^ ^]^ However, with a metal and an insulator whether one reaches equilibrium in the final state is open to debate. It has been known for many years that the contact potential is not enough to explain everything, for instance in the 1957 work of Rose and Ward.^[^ ^89^ ^]^

Why non‐metallic contacts should be more complex follows from standard solid‐state interfacial physics.^[^ ^90^ ^]^ If the contact is a simple ohmic one, it is just a resistor so possibly the separation can be equilibrium. However, if the contact is Schottky and/or there is pinning of the Fermi level at the interface, then the current flow between the two bodies is not the same in the two directions. Both in experiment and modeling, we have found that the changes in the band bending can be complex.^[^ ^76^ ^]^ Furthermore, there can be bulk and surface traps or surface donor states, and these can be sinks for charge and also break reversibility, even for ohmic contacts.

Returning to a point raised earlier, it is not obvious in all cases which of the two boundary conditions of Equations 6 and 7 should be applied or a combination of the two. For instance, for a single contacting asperity as in Figure 2b it is reasonable to use the normal component of the displacement field density for the asperity contact. If the contacting pieces are metallic then there is a step in the electric field at the interface which will be compensated by the free carrier density – Equation 6. Outside the contact there will also be free charges at the surface due to image potentials, the electric fields at the surface are not zero.

For a metal‐insulator contact, the same boundary condition should be relevant at the interface, and similar for the metal away from the contact. What to use for the surface of the insulator away from the contact is less clear and will probably depend upon other factors such as the availability of surface trap states and/or the presence of ionizable or polarizable adsorbates. For two insulators probably the same condition holds at the interface, but this is no longer so clear.

All this points to a major need for more experimental information about systems used for triboelectric studies. Various types of triboelectric experiments, including measurements of triboelectric nanogenerators,^[^ ^91^, ^92^ ^]^ particle impacts,^[^ ^93^, ^94^, ^95^ ^]^ AFM or profilometer contacts,^[^ ^37^, ^76^, ^96^ ^]^ and liquid contacts^[^ ^97^ ^]^ continue to be published. These results are useful, but progress toward a complete understanding of triboelectricity could be hastened by simultaneous measurements of relevant material properties. Without knowledge of the properties of the contacting bodies, many experiments offer only qualitative hints toward theoretical ideas instead of clear and quantitative evidence; modeling can be done, but without sufficient information it can be guesswork. As challenges to the community, we suggest determining the following for all triboelectric experiments:

1. What is the contact potential? That is, for metals, what is the work function? For non‐metals what are the energies of the band(s) involved in charge transfer?
2. What is the crystallography of the contacting surfaces? Both work functions and flexoelectric coefficients are strongly dependent upon these.
3. What is the surface roughness?
4. What are the flexoelectric coefficients, and perhaps also piezoelectric? These can vary by order of magnitude, especially between polymers and ceramics.^[^ ^43^ ^]^
5. What are the dielectric permittivities?
6. What dopants are present, either deliberate or unexpected due to additives and impurities, and surface chemisorbed species as these might be polar?
7. What are the true contact areas, not just the macroscopic ones?
8. What are the elastic constants, and the friction coefficients if sliding is taking place?
9. What are the mobilities for electrons or holes in the materials of interest, perhaps also polar or charged surface species?

Measurements of the work function,^[^ ^98^ ^]^ semiconductor band energies,^[^ ^99^, ^100^ ^]^ permittivity,^[^ ^99^ ^]^ dopant levels,^[^ ^101^ ^]^ surface composition,^[^ ^102^ ^]^ and piezoelectric^[^ ^103^ ^]^ and flexoelectric^[^ ^104^ ^]^ coefficients are standard. As mentioned previously, point seven is often approximated by assuming asperities are just on the edge of yielding,^[^ ^80^ ^]^ though more detailed analysis is possible using AFM,^[^ ^105^ ^]^ and profilometry.^[^ ^106^ ^]^ Elastic constants and friction coefficients are also standard.

Expanding slightly, experiments that will challenge and advance our fundamental understanding are those where single terms are adjusted, and their effects analyzed. As a few plausible examples:

- It is known that temperature changes the flexoelectric coefficients of strontium titanate;^[^ ^107^ ^]^ does the triboelectric charge transfer vary quantitatively in the same way? (Similar coefficient variations have been documented for a relaxor PIN‐PMN‐PT^[^ ^108^ ^]^ and BaTi_1‐x_Zr_x_O_3_.)^[^ ^109^ ^]^ Such experiments would need to track the electromechanical changes as well as elastic constants and tribocharge.
- There is recent data indicating that doping alters apparent flexoelectric coefficients quite dramatically,^[^ ^110^, ^111^, ^112^ ^]^ so it is reasonable that it will similarly change tribocharging. There are already hints on this,^[^ ^113^ ^]^ but more research would be informative. Here the work function and band bending would need to be tracked together with charge transfer and electromechanical coefficients.
- It is known that there can be strong crystallographic dependence of flexoelectric coefficients, for instance a sign reversal for strontium titanate between [100] and [110];^[^ ^65^ ^]^ do these quantitatively map to tribocharge changes?
- Going further afield, what are the flexoelectric (and perhaps piezoelectric) coefficients, work functions, and similar of different types of pharmaceuticals, powders such as grain or flour, desert sand, or volcanic plume material? Do these correlate with the tribocharging?

Returning to what we suggest needs to be determined above, the last one, point 9, extends into a different area of known unknowns, the timescale. In many cases triboelectric experiments are performed on the scale of seconds, and the contact potential differences as well as the potential from electromechanical differences vary on the order of volts per nm. With typical carrier mobilities these numbers suggest very rapid charge transfer in the region of the contact, but outside of this how rapidly charge moves is less clear. There appears to be limited published information on transient behavior in triboelectricity on the important time scales of microseconds or less; more data would move the field forward.

There are also more subtle issues related to polarization accommodation at surfaces or interfaces. It is known that while in theory some surface terminations in materials such as oxides have unbalanced polarizations,^[^ ^48^, ^49^, ^50^ ^]^ in general oxide surfaces rearrange to remove this.^[^ ^51^ ^]^ There are known constraints on the electrostatic stability of insulating surfaces. One of importance herein is the Born effective charge of the atoms, which is the polarization induced by the displacement of each atom. A known constraint is that the sum of the Born charges is zero at a surface,^[^ ^114^, ^115^ ^]^ and how this will couple to deformations at surfaces and interfaces is an open question. (This may also be important for metallic surfaces,^[^ ^116^ ^]^ which is relevant to the boundary conditions.) Delving a little deeper, the discussion herein has almost exclusively been using the classic approach to polarization described in terms of microscopic dipoles, but it would be much better to use what is called the “modern theory of polarization”,^[^ ^117^, ^118^ ^]^ where the quantum mechanical electron density redistribution is included and it is described at a more fundamental level in terms of the Berry phase.^[^ ^119^ ^]^

In summary, the evidence for a central role of polarization and bound surface or interfacial charges in triboelectric contacts appears to be overwhelming. This is established science, and the only relatively new part is the inclusion of flexoelectric contributions which were not widely known until around the start of the 21^st^ century. These play a major role as they automatically lead to significant electromechanical contributions at asperity contacts. However, there remain many uncertainties, particularly with experimental data. Key information needed for modeling is often not measured, and we argue it should be. There is certainly still more to be learned in areas such as the time scale of charge transfer, and more subtle and perhaps important, areas such as surface polarization compensation, which are topics for future work.

## Conflict of Interest

The authors declare no conflict of interest.

## Author Contributions

L.D.M. and K.P.O. contributed equally to this work.

## Data Availability

The data that support the findings of this study are available from the corresponding author upon reasonable request.
